# Long-term experience with a collagen-elastin scaffold in combination with split-thickness skin grafts for the treatment of full-thickness soft tissue defects: improvements in outcome—a retrospective cohort study and case report

**DOI:** 10.1007/s00423-021-02224-7

**Published:** 2021-09-04

**Authors:** Maximilian Lempert, Sascha Halvachizadeh, Clara Charlotte Salfelder, Valentin Neuhaus, Hans-Christoph Pape, Gerrolt Nico Jukema

**Affiliations:** Department of Trauma, University Hospital Zurich, Raemistr. 100, 8091 Zürich, Switzerland

**Keywords:** Matriderm®, Skin grafting, Negative pressure wound therapy (NPWT), Bioburden, Full-thickness wounds, Dermal substitute

## Abstract

**Purpose:**

The management of severe soft tissue injuries to the extremities with full-thickness wounds poses a challenge to the patient and surgeon. Dermal substitutes are used increasingly in these defects. The aim of this study was to investigate the impact of the type of injury on the success rate of Matriderm® (MD)-augmented split-thickness skin grafting, as well as the role of negative pressure wound therapy (NPWT) in preconditioning of the wounds, with a special focus on the reduction of the bioburden.

**Methods:**

In this study, 45 wounds (44 affecting lower extremities (97.7%)), resulting from different types of injuries: soft tissue (ST), soft tissue complications from closed fracture (F), and open fracture (OF) in 43 patients (age 55.0 ± 18.2 years, 46.7% female), were treated with the simultaneous application of MD and split-thickness skin grafting. The study was designed as a retrospective cohort study from March 2013 to March 2020. Patients were stratified into three groups: ST, F, and OF. Outcome variables were defined as the recurrence of treated wound defects, which required revision surgery, and the reduction of bioburden in terms of reduction of number of different bacterial strains. For statistical analysis, Student’s t-test, analysis of variance (ANOVA), Mann–Whitney U test, and Pearson’s chi-squared test were used.

**Results:**

There was no significant difference in the rate of recurrence in the different groups (F: 0%; OF: 11.1%; ST: 9.5%). The duration of VAC therapy significantly differed between the groups (F: 10.8 days; OF: 22.7 days; ST: 12.6 days (p < 0.05)). A clinically significant reduction of bioburden was achieved with NPWT (bacterial shift (mean (SD), F: − 2.25 (1.89); OF: − 1.9 (1.37); ST: − 2.6 (2.2)).

**Conclusion:**

MD-augmented split-thickness skin grafting is an appropriate treatment option for full-thickness wounds with take rates of about 90%. The complexity of an injury significantly impacts the duration of the soft tissue treatment but does not have an influence on the take rate. NPWT leads to a relevant reduction of bioburden and is therefore an important part in the preconditioning of full-thickness wounds.

## Introduction

The management of complex injuries to the extremities with full-thickness wounds poses a challenge to the surgeon. In many cases, the wound will heal with some sort of defects compromising functionality and cosmetics [1]. When primary closure is not feasible, more complex procedures such as split-thickness skin grafts or even fasciocutaneous/microvascular free flaps will be necessary. The split-thickness skin grafts often only provide unsatisfactory results in terms of mechanical stability, flexibility, and cosmetics due to insufficient underlying dermal tissue [2–6], while flap surgery is a more complex procedure with higher risks of complications and morbidity at acceptor side as well as donor side. Moreover, a dedicated plastic surgery department is not widely available in most regional hospitals [2].

Hence, dermal substitutes functioning as a matrix replacing the subcutaneous tissue have been proposed and further developed as a treatment of choice for full-thickness wounds where split-thickness skin grafts must be used [7].

One of those substitutes is Matriderm® matrix (Medskin Dr. Suwelack Skin & Health Care AG, Billerbeck, Germany), which is of bovine origin and provides an elastic and stable neo-dermis formed by a three-dimensional matrix consisting of collagen types I, III, and V, additionally supplemented by elastin hydrolysate [6–8]. The resulting product exhibits a thickness of 1.0 mm, allowing for a single-step combination [4] with an autologous skin graft (nutritional supply by diffusion limiting maximum thickness and distance to the wound base). Within several weeks, this matrix is then replaced by endogenous collagen leading to superior results compared to split-thickness skin grafts alone [6]. There are several reports of successful engraftment in a one-step procedure using Matriderm® and split skin grafting both in vitro [9] and in vivo [3, 10, 11] as well as in clinical trials [12–15].

In complex wounds, conditioning with negative pressure wound therapy (NPWT) prior to definitive closure is often necessary [16]. NPWT involves the delivery of negative pressure through a vacuum pump maintaining a closed environment. NPWT increases perfusion, promotes granulation tissue formation, and reduces edema and the bacterial load [17, 18]. By this, the size of the defect can be significantly reduced, further reducing morbidity at the donor as well as the acceptor side in wounds treated with autologous skin grafting [19]. Furthermore, the reduction of the bioburden through NPWT is an important contribution to the conditioning of wounds leading to improvements in outcome [20]. Additionally, postoperative epicutaneous NPWT has been proven to increase the take rate of skin grafts as it protects the graft from the environment, therefore reducing the risk of infection and detachment. Furthermore, the negative pressure is said to speed up vascularization and thereby further improving the take rate [21].

In our study, we aimed to investigate the impact of the complexity of wounds on the success of Matriderm®-augmented split-thickness skin grafts as well as the role of NPWT in the preconditioning of the defects with a focus on its impact on the development of the bacterial load during the course of the treatment.

## Patients and methods

This monocentric retrospective cohort study was conducted at an academic level 1 trauma center in Switzerland between March 2013 and March 2020. The study was carried out in accordance with the local institutional ethics committee’s terms of reference (Kantonale Ethikkommission Zürich, Switzerland (approval number PB-2016–01,888)). Regular follow-ups were performed at our outpatient clinic usually starting 2 weeks after dismissal of the patient. Follow-up was discontinued at the earliest 1 year posttraumatic unless a patient was lost for follow-up before that. Data collection—based on in hospital electronic documentation systems—begun with admission of the patient and was updated during surgical procedures. Patient-specifics and demographic characteristics as well as type of injury and body region of wound defect were documented. Data was then collected by two authors (ML, CS). We extracted the following parameters:

(i) Patient characteristics (age, gender, comorbidities)
(ii) Injury (location, size, type of injury (soft tissue, fracture, open fracture))
(iii) Treatment (time of NPWT, NPWT changes, duration of stay)
(iv) Microbiology (bacterial growth, antibiotics given)
(v) Outcome variables (rate of recurrence, reduction of bioburden)

The study was conducted and reported according to the STROBE guidelines [22].

Eligibility criteria included an age of over 16 years and performance of Matriderm®-augmented split-thickness skin grafting in a full-thickness wound in a one-step approach with subsequent epicutaneous NPWT for at least 5 days post-surgery and a follow-up period of at least 8 weeks. Patients were stratified into 3 different groups according to the type of injury (fracture; open fracture; soft tissue injury). The standard treatment protocol for wounds, which could not be covered with Matriderm®-augmented skin grafting due to local inflammation or contamination on the day of admission, included multiple surgical debridements, irrigation, and temporary closure with NPWT using a PVA or a PUE foam (Kinetic Concepts Inc. (KCI), San Antonio, TX, USA) prior to the Matriderm®-augmented split-skin-graft procedure. All patients with proven or suspected local infection as well as patients with high risk for infection (e.g., patients with open fractures) were initially treated with an intravenous broad-spectrum antibiotic (mostly amoxicillin/clavulanic acid). Antibiotic therapy was then changed based on the antibacterial sensitivity tests as soon as available. Patients without increased risk for infection (e.g., clean cut wounds) received an antibiotic single-shot prophylaxis (cefazolin) 30 min before every surgical intervention. Microbiological samples were taken during the first debridement, periodically during the sequential interventions, and when definitive closure was performed.

When the wounds showed sufficient granulation tissue and clinical signs of local inflammation (heat, pain, redness, swelling, and loss of function) were absent, surgical coverage of the defects with a combination of Matriderm® matrix and split-thickness skin grafting was performed in a sterile operating theater by an experienced surgeon. Positive bacterial samples without clinical signs of infection did not necessarily lead to delaying definitive wound closure.

## Surgical approach

After preparation of the wound, a Matriderm ® layer of 1.0 mm thickness was carefully cut to fit the size of the defect, with emphasis on avoiding oversizing. Proper positioning ensured full contact to the underlying soft tissue (wound base). Immediate soaking of the matrix indicated adequate placement. After harvesting a skin graft of 0.3–0.5 mm, it was meshed according to the size of the defect (regular 1:1.5 or 1:3) and accurately placed on the Matriderm® layer. The graft was then fixed with single resorbable stitches and covered with a NPWT dressing with a silicon protection layer (Mepithel® (Mölnlycke Health Care AG, Brandstrasse 24, 8952, Schlieren, CH)) in between the mesh graft and the foam dressing. First dressing changes were regularly performed on the fifth day post-surgery. The site of skin graft harvest was covered with a combination of an alginate and polyurethane foam adhesive dressing, which was left in place for 7 days.

The incidence of readmissions of included patients due to local complications, which required revision surgery, was recorded. Furthermore, the time between first debridement and wound closure as well as number of interventions prior the wound closure was recorded. Special focus was further put on length of stay (LOS) and the development of the bacterial spectrum. Additionally, the presence of diabetes mellitus, peripheral vascular disease (PVD), smoking, alcohol/drug abuse, and an immunodeficiency (human immunodeficiency virus (HIV) and malignancy) or immunosuppressive medication was noted, as they might impact the wound healing process.

## Statistical analysis

Continuous data are presented with mean ± standard deviation (SD). Categorical variables are summarized as number and percentages. Normally distributed continuous variables were compared using Student’s t-test; group comparisons with more than two groups were performed with ANOVA. Skewed distributed comparisons were performed using Mann–Whitney U test. Pearson chi-square test was used for comparison of groups on discrete variables. The statistical analyses were performed using R (R Core Team (2018). R: A language and environment for statistical computing. R Foundation for Statistical Computing, Vienna, Austria. URL https://www.R-project.org/.)

## Results

Between December 2012 and March 2020, we treated 43 patients with a total of 45 soft tissue defects at our level 1 trauma center with Matriderm®-augmented split skin grafting in a one-step approach. Out of 45 treated defects, 44 (97.7%) were located on the lower extremity. The demographics of our study are depicted in Table 1.

**Table 1:** Demographics

n (cases)	45
Age (mean (SD))	54.4 (18.72)
Female (%)	21 (46.7)
Closed fracture (%)	6 (13.3)
Open fracture (%)	18 (40.0)
Soft tissue (%)	21 (46.7)
Gustillo-Anderson classification of open fractures	
II	3 (16.7)
IIIA	10 (55.6)
IIIB	1 ( 5.6)
IIIC/amputation	4 (22.2)
NPWT (%)	
No	2 (4.4)
Instill	7 (15.6)
Regular	36 (80.0)

We screened all patients for comorbidities. Fifteen patients (34.8%) had none, while 28 patients (65.1%) had one to three relevant comorbidities. There was no significant difference between the three injury groups, nor did we find a relevant impact on the rate of recurrences.

The number of recurrences according to type of injury is shown in Table 2. In 39 (90.7%) out of 43 patients, we could achieve sufficient wound closure with the first Matriderm®-augmented skin graft. The closed fracture group had no reoccurrence of the treated defect, while both the open fracture group and the soft tissue group both had recurrences in 2 patients, who required revision surgery.

**Table 2:**
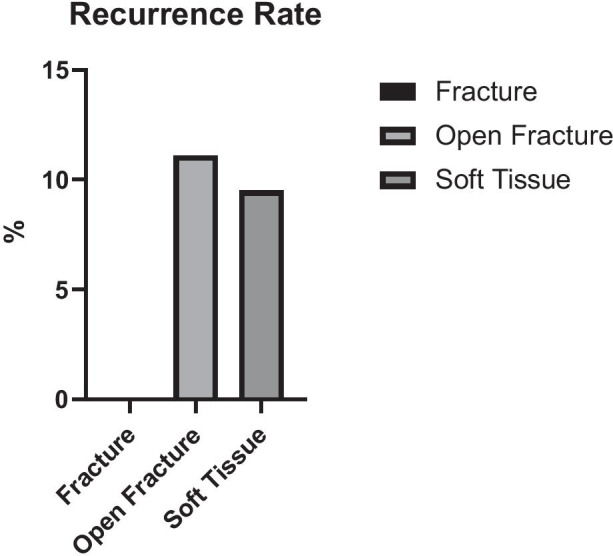
Rate of recurrence stratified to injury type

Forty-three out of 45 (95.5%) wounds (cases) were treated with NPWT for variable periods of time before the performance of Matriderm®-augmented split skin grafting. We stratified the duration of treatment according to three different types of injuries. Cases with open fractures required NPWT significantly longer than those with closed fractures or soft tissue defects without osseous involvement prior to the closure of the defect (Table 3). Furthermore, those cases being treated with V.A.C Ultra™ (V.A.C. Instillation™) therapy with diluted polyhexamethylene biguanide (PHMB) solution required NPWT significantly longer than those receiving regular NPWT (13.9 vs. 20.6 days).

**Table 3:**
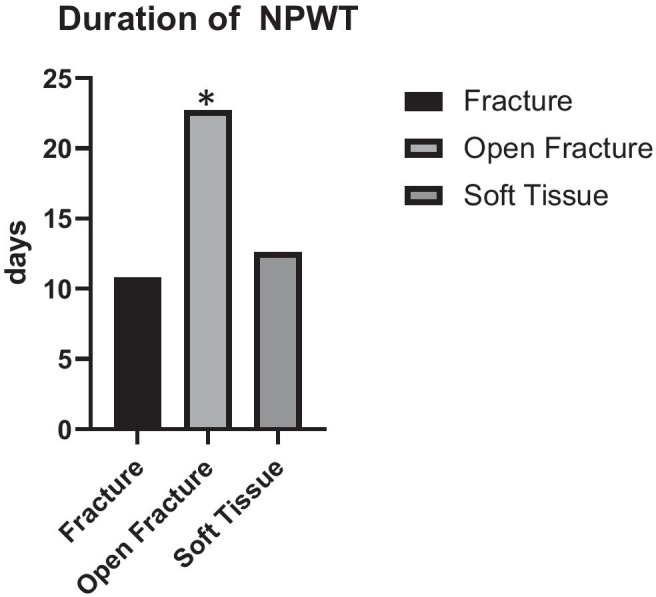
The mean duration of NPWT therapy stratified to injury type (p < 0.05)

The number of VAC—changes before the Matriderm@-augmented split skin grafting procedure stratified to injury pattern—differed significantly between the different types of injuries. Patients with open fractures underwent a mean of 4.9 changes while the ones with closed fractures underwent 2.5 and those with only soft tissue injuries 2.9 procedures (p < 0.05) until the final wound closure with Matriderm®-augmented split skin grafting was performed (Table 4).

**Table 4:**
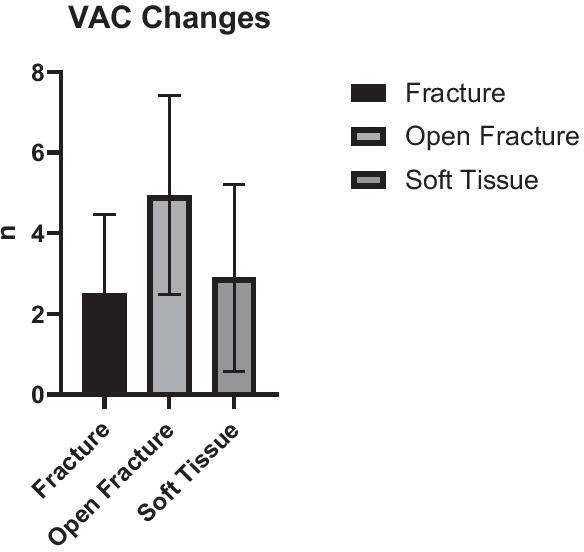
Number of VAC changes prior to definitive wound closure

The number of changes in the antibiotic treatment in relation to the injury type also showed clear differences. Patients with open fractures received a mean of 3.2 different antibiotic substances compared to 3 in the fracture group and 2.7 in the soft tissue group.

The length of stay was also impacted by the type of injury causing the treated wound defect. Patients with underlying open fractures were hospitalized for 48.2 days compared to 43.5 days in the soft tissue group. Patients with underlying closed fractures were hospitalized for 29.5 days on average (Table 5).

**Table 5:**
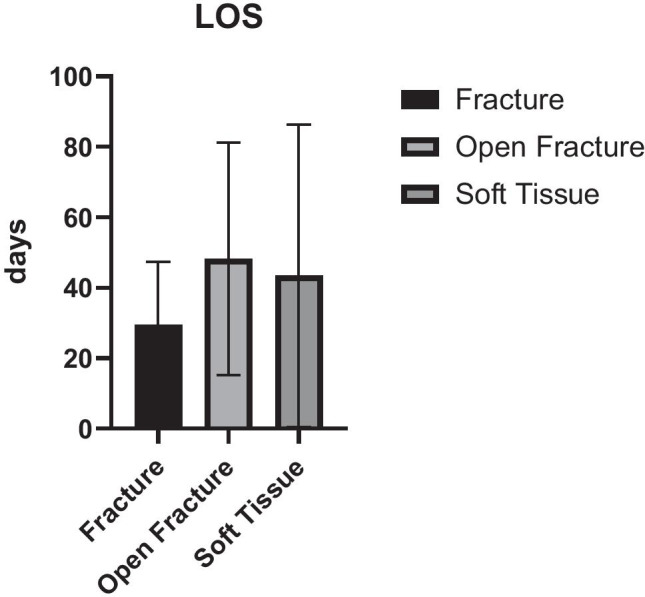
Length of stay stratified to injury pattern

When comparing the bacterial load found in the first debridement of the wounds and the bacterial load on the day of definitive wound closure, a clear trend of reduction can be seen (Table 6).

**Table 6:** Bacterial shift stratified to injury type

	F	OF	S	p
n_bac_first (mean (SD))	2.33 (1.97)	1.38 (1.59)	1.94 (2.36)	0.552
n_bact_md (mean (SD))	0.50 (0.58)	0.33 (0.71)	0.30 (0.67)	0.88
bact_shift (mean (SD))	− 2.25 (1.89)	− 1.90 (1.37)	− 2.60 (2.22)	0.704

## Case report

A 24-year-old man sustained a severe crush injury to the right lower limb in a forklift accident. The patient presented with an open femur fracture (Gustilo-Anderson Type IIIC, Picture 1) with severe comminution and subtotal amputation of the thigh and severe damage to the sciatic nerve. Furthermore, he sustained a substantial soft tissue defect in the right gluteal area with a foreign body in situ. The CT-angiogram showed a traumatic disruption of the distal superficial femoral artery. Subsequently, the patient developed an ischemic compartment syndrome in the right lower leg and foot that required fasciotomy.

**Picture 1:**
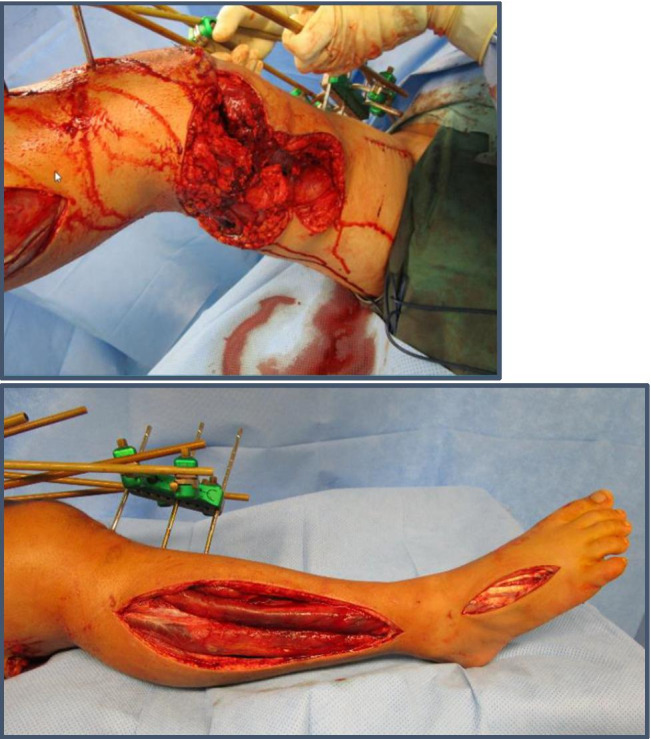
**a** Left side and **b** right side: bilateral dermatofasciotomy of the lower leg and foot

On the day of admission, we performed the implantation of an Omniflow® (LeMaitre Vascular, Inc., Burlington, MA, USA) vascular prosthesis (6 mm) from the disrupted superficial femoral artery to popliteal artery (end-to-end) with external fixator fixation of the fracture with shortening of the femur for 4 cm to reduce the tension of the soft tissue, including the sciatic nerve. Bilateral dermatofasciotomy of the lower leg and foot was performed in combination with debridement of the bone and soft tissue. After an extended lavage, NPWT for temporary occlusion of all wounds was applied. The soft tissue defect on the medial side of the thigh resulting from the OF was treated with regular NPWT for a total of 31 days. The vacuum dressings were consecutively changed (a total of eight times) in the operating theater in combination with recurrent lavage and surgical debridement. We applied white PVA dressings to the wound and covered them each time with a second foam layer on top of black PU dressings. After 31 days of NPWT, the defect was covered in a one-step procedure with 1-mm MD augmentation and a split-thickness mesh graft. The bacterial cultures of the patient showed multiple bacterial specimens (*Stenotrophomonas maltophilia*, *Bacillus cereus*, and *Staphylococcus epidermidis*), which we treated according to the bacterial sensitivity with various combinations of different antibiotics (amoxicillin/clavulanic acid, clindamycin, vancomycin, and levofloxacin). When the split-thickness skin graft was performed, the patient had two clean swabs from the previous dressing changes in a row and was under oral antimicrobial therapy with levofloxacin, which was applied as suppression therapy for a total of 442 days (Picture 2).

**Picture 2:**
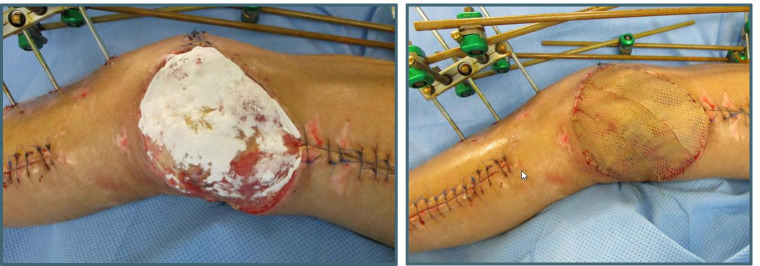
**a** Left side and **b** right side: application of Matriderm® 1 mm in combination with mesh graft transplantation

The MD-augmented mesh graft showed excellent healing with good functionality, stability, and elasticity (Picture 3).

**Picture 3:**
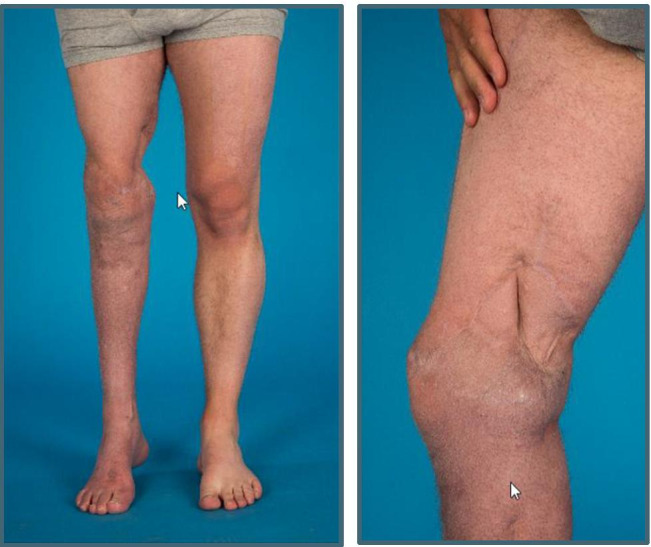
**a** Left side and **b** right side: right leg 2.5 years after mesh graft transplantation with Matriderm™ augmentation

The soft tissues, which were covered 3.5 years prior with mesh graft and MD augmentation, continued to be intact and free of irritation with good functionality until today.

## Discussion

To our best knowledge, we report one of the largest cohorts of soft tissue defects treated with split skin grafts augmented with a non-cross-linked collagen-elastin matrix (Matriderm®) for wound closure. In our clinic, we achieved promising results with the combination of split skin grafts and this dermal substitute in a one-step approach in complex soft tissue defects, which is in accordance with the current literature. We could achieve wound healing in 90.7% of the patients with the first approach, which is in accordance with the findings of other groups [3, 10, 11]. We had a total of 5 recurrences of soft tissue defects (11.1%) in 4 patients (9.3%). In one of those cases, there was an ongoing septic arthritis of the ankle joint after a grade III open fracture, 4 weeks after the injury and 3 weeks after the augmented mesh graft procedure. Due to insufficient healing after the Matriderm®-augmented mesh graft, this patient required vascular free flap surgery for permanent closure of the defect, and due to the ongoing osteomyelitis of the ankle joint, subsequently an arthrodesis of the joint became necessary. The second case, a patient with severe rheumatoid arthritis, presented with a reoccurring wound defect on the left lateral malleolus in a chronic osteomyelitis 3.5 years after the first Matriderm®-augmented mesh graft procedure. A first attempt of revision with Matriderm@-augmented skin grafting failed, and the patient subsequently underwent free flap surgery with good long-term outcome. The third case was a young woman with a severe soft tissue trauma to the left thigh sustained in a truck accident. She needed revision of the augmented skin graft in a small fraction of the initially treated defect about 5 months after the first surgery. She subsequently fully recovered. The last patient, who required revision surgery, suffered traumatic amputations of both lower legs. He presented with recurrences of the treated wounds after 10 months on the right leg and 34 months postoperatively on the left leg, respectively, which both had to be treated with free flap surgery. While the right stump showed a good long-term result, the left stump developed ulcers after flap surgery, which required long-lasting wound treatment by out plastic surgery department, which is still ongoing (Winter 2020).

We applied different NPWT systems depending on the complexity of the defects. Less complicated defects were treated with standard NPWT while more complex defects received instillation NPWT (VAC Ultra®). The reasoning behind this approach was the assumption of a greater bioburden in more complex wounds such as open fractures or obviously contaminated wounds, therefore aiming to reduce the bioburden by continuous irrigation [17] in addition to surgical debridement. In our opinion, the combined procedure with repetitive surgical debridement and negative pressure therapy is more efficient and gentler than surgical debridement alone. NPWT stimulates wound healing by improvement of vascularization of the wound bed. The complexity of the wounds is also reflected in the duration of VAC therapy with open fractures requiring NPWT significantly longer compared to closed fractures or soft tissue defects without osseous involvement subsequently leading to longer periods of hospitalization as well. Also, when comparing the number of VAC changes and surgical debridements required prior to the skin grafting, patients with open fractures required the most procedures before the application of Matriderm®. This staged approach aimed at conditioning even the most complex wounds in a way that closure with Matriderm®-augmented skin grafting would become possible in a one-step approach. In addition to radical and sufficient surgical debridement, NPWT played a major role in the preconditioning of wounds as it increases perfusion within the wound, accelerates granulation tissue formation, and reduces edema as well as the bioburden [18]. With this approach, we achieved healing after the first Matriderm®-augmented split skin grafting in almost 90% of the cases, even in complex wound situations after open fractures. Those positive results go hand in hand with those of other research groups, where the augmentation with Matriderm® also lead to an improvement in the outcome after the treatment of soft tissue defects with low rates of recurrences [2, 21, 23].

With the combination of debridement and NPWT prior to the skin grafting, we could further achieve a relevant reduction of the bacterial load in the treated defects of all three different injury groups, which is in accordance with the results of other groups [20, 24–29]. But not all defects were free of pathogens when we performed the skin grafting augmented with Matriderm®, which is in stark contrast to the methods described by other studies [2, 30]. In nine cases, we performed the final wound closure in wounds, which were not yet free of pathogens, what did not seem to have a negative influence on the healing as those patients showed good long-term results with a reduced duration of hospitalization and number of surgical procedures.

The complexity of the wound also influenced the number of different antibiotic substances given to the patients, with those with open fractures receiving the highest number of different antibiotics.

A surprising result was that the existence of comorbidities did not have a relevant impact on the outcome, which contrasts with the results of other groups [31, 32]. The main reason for this discrepancy can be found in the different severities of injuries studied. While we mainly treated patients with locally limited injuries, the studies mentioned above investigated patients with systemic impairments due to injuries.

The great advantages of Matriderm® as a product compared to other dermal regeneration substitutes such as the dermal regeneration template Integra® (Integra LifeSciences, 1100 Campus Road, Princeton, NJ, 08,540) are first of all the possibility of a one-step approach [2, 23] and a higher graft take rate [21], which leads to shorter duration of hospitalization and a reduction of surgical procedures required. Also, compared to much more complex and time-consuming procedures promoting dermal regeneration such as keratinocytes cultures [33], Matriderm® is our preferred choice due to time efficiency, costs, and functional outcome. This also strongly promotes the economic benefit of the application of Matriderm® despite its costs [34] compared to well-established but long-lasting and complex treatments such as flap surgery, which are associated with relevant donor and acceptor side morbidity [35]. Flap surgery can result in flap necrosis [36–38] as well as relevant morbidity at the donor side such as scaring [5] making it a highly delicate procedure requiring experienced microsurgeons. Lack of experienced surgeons or the wound conditions themselves may make flap surgery not feasible in multiple cases—just as discussed in our case report—where due to vascular injuries, we had to abstain from flap surgery and resort to Matriderm®-augmented split-thickness skin grafting after close consultation with our colleagues of the department of plastic surgery. But of course, we are aware of the limitations of our Matriderm®-based approach and that there are plenty of indications making flap surgery mandatory with excellent results in terms of functionality and cosmetics [39, 40]. In our experience, Matriderm®-augmented split-thickness skin grafts achieve high stability and elasticity of soft tissue coverage, which is at the outmost importance in situations such as amputation stump coverage or in defects close to joints. Furthermore, we found—in accordance with other studies—the scarring to be much smoother compared to split-thickness skin grafting alone with clinically significantly less postoperative development of keloids, leading to a higher overall patient satisfaction. Ryssel et al. compared autologous skin grafts to a combination of Matriderm® matrix and autologous skin grafts. The combination led to a significantly increased elasticity of the newly formed skin after wound healing [2, 23, 35]. Furthermore, Min et al. showed that areas covered with Matriderm® showed similar qualities in terms of skin barrier and skin color to those of the adjacent normal skin [23], similar results were presented by Watfa et al. also reporting positive results concerning sensory function after augmentation of harvest side in transgender surgeries with Matriderm® [41]. Synoptically, we therefore think that Matriderm®-augmented split skin grafting offers a valid treatment option in many different cases of full-thickness wounds and that its indications should be expanded and that it should be more frequently used as an adjunct to flap surgery in the future, always keeping its limitations in mind.

Our retrospective cohort study does have limitations. The main limitation is the fact that our study is not of a comparative nature. Therefore, we are not able to demonstrate the difference between Matriderm®-augmented mesh grafts and mesh grafts without augmentation. But in the current literature, it is clearly demonstrated that complex traumatic wounds with soft tissue defects are frequently showing complications in wound healing after regular mesh graft procedures without Matriderm® augmentation. Furthermore, we included different kinds of wounds and classified them as “complex.” But there is no practical classification system for the complexity of full-thickness defects, and therefore, we based the inclusion of cases on our clinical experience. Nevertheless, we think that our report of healing rates of about 90% of cases after Matriderm®-augmented split skin grafting is still of clinical and scientific benefit.

The main strength of our study is its large cohort of traumatic soft tissue defects, which is to our best knowledge one of the largest in the current literature. Furthermore, our investigations are not only limited to the skin grafting procedure alone but also involve the process of wound conditioning prior to the grafting. Moreover, our study covers an extensive follow-up period, which allows well-founded statements regarding long-term results.

## Conclusion

We could demonstrate in this study that Matriderm®-augmented split-thickness skin grafting leads to generally good long-term results even in complex wounds with a relevant bioburden. The complexity of the wounds in terms of injury pattern and bioburden did not have a significant impact on the long-term results. In order to achieve the best possible outcome, different aspects of a wound defect must be taken into consideration and an interdisciplinary approach with the involvement of trauma, orthopedic, and plastic and vascular surgeons as well as interventional radiologist is often required. With an individual approach to each wound in terms of wound conditioning using NPWT as an adjunct to debridement as well as adequate antibiotic treatment, Matriderm®-augmented split skin grafting allows wound closure in a timely and economically highly efficient way even in extremely complex defects. Further studies of comparative nature between augmented and non-augmented skin grafts as well as between Matriderm®-augmented skin grafting and flap surgery are needed to further investigate the role of Matriderm®-augmented split-thickness skin grafting in complex wounds.
